# Carotenoid profiling of the leaves of selected African eggplant accessions subjected to drought stress

**DOI:** 10.1002/fsn3.370

**Published:** 2016-04-18

**Authors:** Elias K. Mibei, Jane Ambuko, James J. Giovannoni, Arnold N. Onyango, Willis O. Owino

**Affiliations:** ^1^ Department of Food Science and TechnologyJomo Kenyatta University of Agriculture and TechnologyNairobiKenya; ^2^ Department of Plant Science and Crop ProtectionUniversity of NairobiNairobiKenya; ^3^ Boyce Thompson Institute for Plant ResearchCornell University CampusIthacaNew York14853

**Keywords:** African eggplants, Carotenoids, chlorophylls, drought stress, stress tolerance

## Abstract

African eggplants (*Solanum aethiopicum* and *S*. *macrocarpon*) are among the most economically important and valuable vegetable and fruit crops. They are a major source of biologically active nutritional substances and metabolites which are essential for plant growth, development, stress adaptation and defense. Among these metabolites are the carotenoids which act as accessory pigments for photosynthesis and precursor to plant hormones. Though African eggplants are known to be resistant to various abiotic stresses, the effect of these stresses on secondary metabolites has not been well defined. The objective of this study was to establish the effect of drought stress on carotenoid profiles of nineteen African eggplant accessions selected based on leaf and fruit morphological traits. Stress was achieved by limiting irrigation and maintaining the wilting state of the crops. Fresh leaves were sampled at different maturity stages; before stress, 2 weeks and 4 weeks after stress for carotenoid analysis. The fresh harvested leaf tissues were immediately frozen in liquid nitrogen and ground. Analysis was carried out using a Dionex HPLC machine coupled to Photo Array Detector and Chromeleon software package (Thermo Fisher Scientific Inc, Waltham, Massachusetts, USA). Major carotenoids viz;. Xanthophylls (neoxanthin, violaxanthin, zeaxanthin and lutein) and carotenes (*β*–carotene and *α*–carotene), phytofluene, lycopene, phytoene as well as chlorophylls (chlorophyll‐b and Chlorophyll‐a) were targeted. The carotenoids increased with maturity stage of the crop. Although the stressed crops reported significantly decreased amount of carotenes, chlorophylls, neoxanthin and violaxanthin, the concentration of zeaxanthin increased with stress whereas lutein had no significant change. Chlorophyll‐a was significantly high in all the control accessions. Two accessions reported significantly higher contents of carotenoids as compared to the other accessions. The results of this study indicate that water stress has significant impact on the concentration of some carotenoids and photosynthetic pigments. This will definitely add value to the study of stress tolerance in crops.

## Introduction

African eggplants (*Solanum aethiopicum* and *S*. *macrocarpon*) are among the nutritionally important and valuable crops in the Solanaceae family (Chadha and Mndiga 2007). They constitute important fruit and leaf vegetables in Africa (Shippers 2000) due to their dual value: leaves are used as cooked vegetables and the fruits are also edible. The leaves are appreciated for their slightly bitter taste and are eaten separately or in sauces. The fruit flesh on the other hand can be sweet or bitter in taste. The bitter cultivars have been used as medicine in many African countries (Chadha and Mndiga 2007). Besides, there is also increasing evidence that the intake of their leaves and fruits have favorable impact on the incidence of many chronic diseases including diabetes (Kwon et al. 2008). In addition, most indigenous vegetables have been reported to be rich in micronutrients and nutritional components (Mibei et al. 2011) and phytochemicals including alkaloids, flavonoids, tannins, saponins, steroids, phenols and antioxidants (Mibei and Ojijo 2011; Mibei et al. 2012). These are of health or nutraceutical significance therefore, authenticates their usefulness for medicinal purposes (Briskin 2000).

Despite the importance of many indigenous plants, stress has been reported as a major limiting factor leading to change in their growth and development thus disrupting metabolic homeostasis. This affects plants and requires an adjustment of metabolic pathways for acclimation (Suzuki et al. 2012). Metabolomics is an essential part of a systems biology approach to study plant defense, since different metabolic profiles are indicative of changes in metabolic pathways (Hankemeier 2007). Therefore, when plants are subjected to water stress, they change physically and chemically in numerous ways. In addition, they produce a huge number of metabolites to adapt to the stress conditions. Among these metabolites of interest are the carotenoids which are widely distributed in nature. They not only act as accessory pigments for photosynthesis and as precursors to plant hormones (Cazzonelli 2011), but also impart various benefits to human health (Johnson 2002; Rao and Rao 2007). They are potent antioxidants and free radical scavengers (Grassmann et al. 2002). This is believed to contribute to their ability to modulate the pathogenesis of coronary cancers (van Poppel and Goldbohm 1995) and coronary heart disease (Kritchevsky 1999). On the other hand, lycopene intake is associated with a decreased incidence of prostate cancer (Giovannucci 2002) and diabetes (Facchini et al. 2000).

Due to their importance in diet and health benefits, carotenoids have been extensively studied in different matrices to analyze their distribution and levels in plants. Based on this, the metabolic adjustments in response to the water stress conditions may be analyzed and this will highlight carotenoids that play important roles in metabolism and physiology of the plant. These carotenoids as well are important for human health as they have nutritional and medicinal properties.

## Materials and Methods

### Plant material

Seeds of seventy four African eggplant accession were obtained from the from local farmers, farmer groups and a variety of gene banks at local and regional centers and institutes which include, Kenya Agricultural Research Institute (KARI), Muguga Kenya and the Asian Vegetable Research Development Centre (AVRDC), Arusha, Tanzania.

Nineteen African eggplants accessions were selected (Table 1) based on their morphological traits. The traits were based on fruit size and weight, fruit shape, fruit length, flower color, leaf blade length and width. The selected accessions were grown alongside each other in the greenhouse at the Boyce Thomson Institute for Plant Research, Cornell University, USA during March ‐ May, 2015 under carefully controlled and optimal growth conditions.

**Table 1:** List of selected African eggplants from the accessions provided by AVRDEC‐ESA

S/no	RVI code	Genus	Species	Name
1	RVI00343	*Solanum*	*macrocarpon*	CN012
2	RVI00199	*Solanum*	*sp*	EX‐DAR
3	RVI00201	*Solanum*	*aethiopicum*	GKK‐AE‐158
4	RVI00332	*Solanum*	*aethiopicum*	RNL187‐194
5	RVI00271	*Solanum*	*aethiopicum*	Line 87
6	RVI00445	*Solanum*	*sp*	S0004
7	RVI00333	*Solanum*	*aethiopicum*	SANGAWILI
8	RVI00259	*Solanum*	*aethiopicum*	Line 55
9	RVI00265	*Solanum*	*aethiopicum*	Line 21
10	RVI00273	*Solanum*	*aethiopicum*	Line 89
11	RVI00511	*Solanum*	*aethiopicum*	SENGEREMA 1
12	RVI00432	*Solanum*	*sp*	N4
13	RVI00246	*Solanum*	*aethiopicum*	Line 112
14	RVI00328	*Solanum*	*aethiopicum*	LOCAL MALI
15	RVI00327	*Solanum*	*aethiopicum*	AUBERGINE BLANCHE
16	RVI00342	*Solanum*	*aethiopicum*	OFARIWA'A
17	RVI00330	*Solanum*	*aethiopicum*	Local Gaya
18	GBK 050591	*Solanum*	*aethiopicum*	Kenya
19	RVI00438	*Solanum*	*aethiopicum*	MM1308

RVI‐Accession registration code used in AVRDEC, sp ‐ species.

### Treatments

The African eggplant seeds were germinated in the greenhouse in trays and the seedlings transplanted after 4 weeks of germination. Normal irrigation was maintained before and 5 days after transplanting of the seedlings to keep the soil moisture at over 90% field capacity. The seedlings (one per pot) were grown in 15 cm‐diameter pots containing growth media using randomized complete block design with three replications. The experiment had two treatments; water stress and control experiments. Water stress treatments were initiated after 5 days of transplanting. This was achieved by stopping irrigation for a few days and soil moisture monitored every day using Delmhorst model KS‐D1 Digital Soil Moisture Tester (Delmhorst Instrument Co., Towaco, NJ). The wilting state of the crops was maintained and losses in soil moisture below 60% represented transpiration and evaporation. Therefore irrigation was done after every 2 days with equal amount of water (approximately 1 L) to compensate for this. For the control treatment, continued watering with sufficient amount of water (normal irrigation) was maintained throughout.

### Chemicals

Tetrahydrofuran, petroleum ether, ethyl acetate, methanol and methyltert‐butyl ether were purchased from Fischer Scientific (Fair Lawn, NJ). All reagents were analytical or HPLC grade. All mobile phases and samples were filtered before use.

### Sample collection

Fresh leaves were sampled early in the morning at different growth and development stages (before stress, 2 weeks and 4 weeks after stress) for carotenoid analysis. The materials were harvested from equivalent fully expanded leaves from each plant at each stage. The harvested leaf tissues were immediately plunged (snap‐frozen) in liquid nitrogen to quench further metabolism. Afterwards, they were ground in liquid nitrogen and stored in 15 mL falcon tubes at −80°C. The frozen leaf tissues were later used for carotenoid analysis by HPLC.

### Carotenoid extraction

Carotenoids were extracted from the frozen leaf tissues using a modified protocol from Alba et al. (2005). About 200 mg of each tissue was weighed into 2 mL Eppendorf tubes and 2 beads added. Fifty microliters (50 *μ*L) of 3 mg/mL magnesium carbonate suspension was added to each tube and 300 *μ*L of tetrahydrofuran (THF) added to each tube. The mixture was homogenized in FastPrep machine (FastPrep‐24, MP Biomedicals, Santa Ana, California, USA) (45 sec, speed = 5.0) and incubated at 4°C (in ice) for 20 min in dark. 300 *μ*L of methanol was also added and homogenized then incubated at 4°C for 10 min. The homogenate was then transferred to Spin‐X filter, centrifuged for 1 min at 1487 × g (4°C). 150 *μ*L of THF and 150 *μ*L of methanol were added to original extraction tube and vortexed. 1 mL pipettor (cut tip) was used to transfer all THF/methanol/debris to spin‐X filter and centrifuged again. The filtered extract was then transferred to new 2 mL tube and 450 *μ*L of THF added to debris pellet in spin‐X filter and incubated on ice for 15 min (dark); centrifuged for 5 min at maximum speed. The filtered extracts were combined and 375 *μ*L petroleum ether and 150 *μ*L of 25% NaCl were added to each combined extract and vortexed vigorously. It was then centrifuged at maximum speed at 4°C for 3 min to separate phases and the upper phase transferred to new 2 mL tube. The interphase/lower phase was re‐extracted with 500 *μ*L petroleum ether and the upper phases removed and combined with like samples. The petroleum ether extract was then rotor evaporated for 20 min at 45°C (to near dryness). When HPLC was not conducted immediately, the dried extracts were stored under nitrogen (N_2_) at −80°C (dark). 500 *μ*L ethyl acetate was added and incubated at room temperature for 15 min to resuspend carotenoids; vortexed well and carotenoid suspension filtered through 0.45 *μ*m nylon syringe filter (Cameo 3N syringe filter, GE Water and Process Technologies Trevose, Pennsylvania, USA).

### HPLC analysis (YMC C_30_ column)

Carotenoid analysis was carried out using a Dionex HPLC machine (P680 HPLC pump, ASI‐100 Automated Sample Injector; PDA‐100 Photo Array Detector) and Chromeleon (v6.40 software package). Carotenoids were separated with a polar to non‐polar gradient (0–5 min 100% methanol:0.1% ammonium acetate; 6–25 min ramp to 4% methanol:0.1% ammonium acetate and 96% methyl t‐butyl ether; 26–30 min ramp to 100% methanol:0.1% ammonium acetate; 31–35 min 100% methanol:0.1% ammonium acetate) through a guard cartridge (YMC Carotenoid S‐5, 4.0 mm × 20 mm DC guard; Waters), C30 column (YMC Carotenoid S‐5, 4.6 mm × 250 mm; Waters) assembly. Five channels were used for data acquisition: channel 1 (286 nm); channel 2 (348 nm); channel 3 (434 nm); channel 4 (450 nm) and channel 5 (471 nm).

### Identification and quantification of carotenoids

Peak identification was performed as described in Alba et al. (2005). The carotenoids were identified by comparing their retention time and spectra with respective authentic standards analyzed under identical analytical conditions. Peak areas of each of the standards were used to draw the standard curve and quantify the carotenoids.

### Data analysis

The data were subjected to the statistical analysis of the variance (ANOVA) to evaluate significant differences between the different developmental stages and treatments of African eggplants. The analysis was performed using GenStat discovery 14th Edition (Payne et al. 2011) at 5% level of significance. Mean separation was done by Fisher's protected least significant difference (LSD) test using GenStat at *P* = 0.05.

## Results

**Figure 1:**
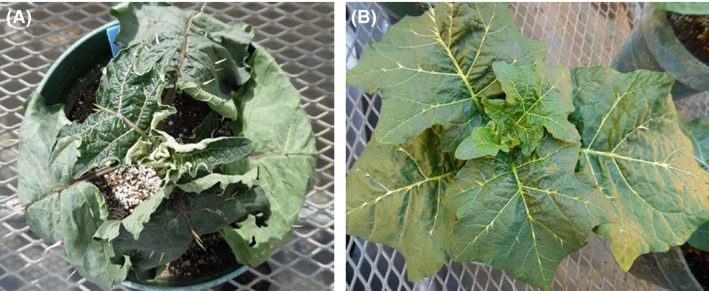
The picture of African eggplant (RV100332) accession (A) Water stressed (B) Control treatments.

The difference in plant morphology under stress is illustrated in Figure 1.

### Carotenoids profile of African eggplant leaves

The HPLC fingerprinting of the African eggplant leaf extracts revealed the presence of the carotenoids such as neoxanthin, violaxanthin, zeaxanthin, *α*‐carotene, *β*‐carotene, lutein and other unknowns. The chlorophylls (chlorophyll a and chlorophyll b) were also identified (Table 2). Phytoene, phytofluene and lycopene were not detected.

**Table 2:** Carotenoids separated on a reverse‐phase C30 HPLC system and spectral characteristics used in identification from photodiode array detection

Carotenoid	Spectral characteristics(nm *λ*_max_)	Retention time(min)
Neoxanthin	466	6.99
Violaxanthin	433	7.68
Chlorophyll b	465, 471	10.6
Phytoene 1	284–286	~10.8
Lutein	442, 450	~11
Chlorophyll a	430	12.73
Zeaxanthin	430	13.33
Phytoene 2	284–286	~13.5
Phytofluene 1	343/348	~14.3
Phytofluene 2	343	~15.1
Others (unknown)	441, 443	15.96, 16.44
*α*‐Carotene	407	17.03
*β*‐carotene	450	~17.1, 17.6
Cis‐Lycopene	471	~22.2
Trans‐Lycopene	471	~24

**Figure 2:**
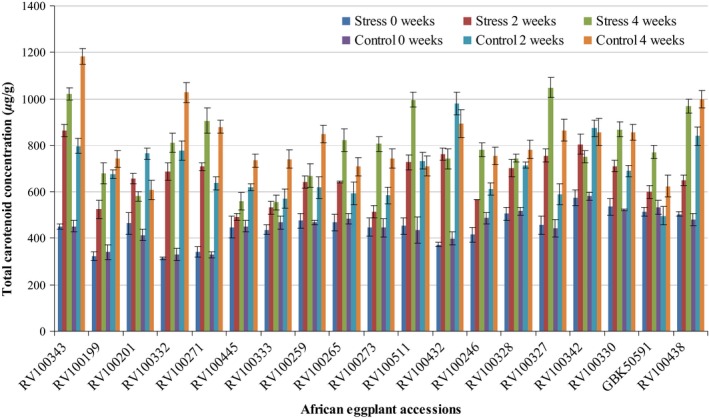
The total carotenoid concentration in the leaves of African eggplant subjected to stress and control at different growth and development stages; 0 weeks, 2 weeks and 4 weeks. Total carotenoid contents were calculated by taking the sum of each compound in all African eggplants. The results are presented as *μ*g/g ± SD fresh weight.

The total carotenoids of the leaves of the selected African eggplants subjected to drought stress and controls are reported in Figure 2. The concentration increased with the growth stages of the African eggplants with 4 weeks sampled leaves recording significantly higher amount (*P* < 0.05). The highest of the estimated carotenoids in mature leaves was reported in RV100327 (1049.4 ± 43.7 *μ*g/g FW), RV100343 (1020.9 ± 26.9 *μ*g/g FW), RV100438 (969.7 ± 30.3 *μ*g/g FW) and RV100511 (996.2 ± 32.3 *μ*g/g FW) stressed accessions whereas for the control RV100343 (1182.3 ± 33.6 *μ*g/g FW), RV100332 (1027.2 ± 43.9 *μ*g/g FW) and RV100438 (998.9 ± 37.6 *μ*g/g FW) accessions had considerable high concentration of the total carotenoids. Unlike the other accessions, the concentration of the total carotenoids for GBK50591, RV100271, RV100265, RV100511, RV100327, RV100330 and RV100199 significantly increased (*P* < 0.05) in stressed crops as compared to the controls. Contrary to the trend for the other accessions, RV100201, RV100432, and RV100342 reported significantly higher (*P* < 0.05) carotenoids at 2 weeks then the content reduced as the plant grows at 4 weeks.

During the growth of the plant, increase in the carotenoids occurred in almost all the accessions. This was evident in the leaves sampled after 4 weeks (Fig. 2) reporting higher contents as compared to other stages. The chlorophyll contents of the leaves were also significantly higher (*P* < 0.05) as compared to the carotenoids. Zeaxanthin and *α*‐carotene on the other hand reported low concentrations in both the stressed and control plants.

**Figure 3:**
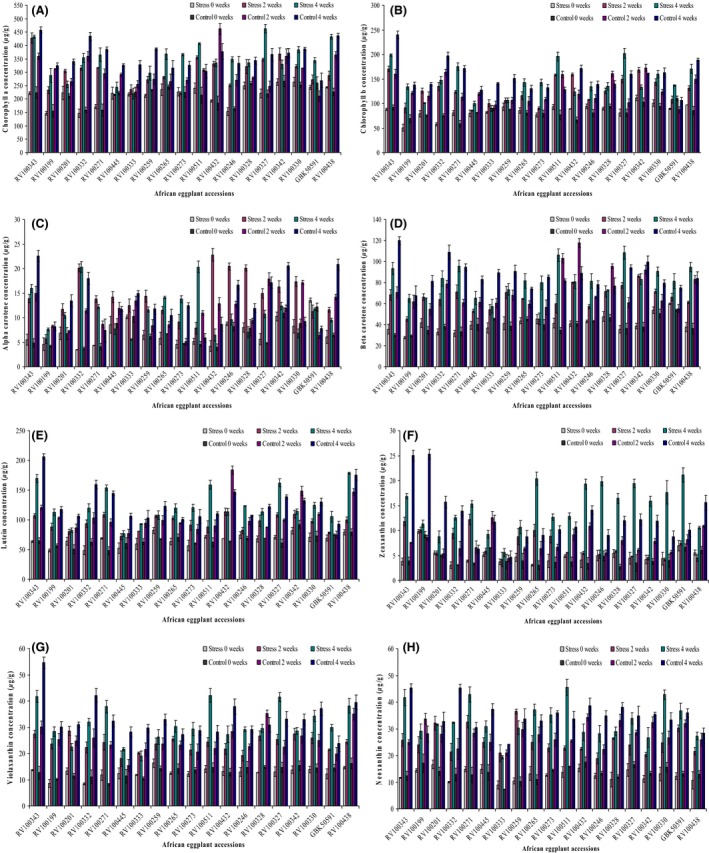
The carotenoid and chlorophyll concentration of different accessions expressed as *μ*g/g ± SD fresh weight (*n* = 3) at different growth and development stages (0 weeks, 2 weeks and 4 weeks) for stress and control treatments. (A) Chlorophyll a, (B) chlorophyll b, (C) alpha carotene, (D) beta carotene, (E) lutein, (F) zeaxanthin, (G) violaxanthin and (H) neoxanthin.

The individual carotenoid and chlorophyll concentrations of each African eggplant accession are shown in Figure 3. The concentration of the different carotenoids varied within between the different accessions.

**Figure 4:**
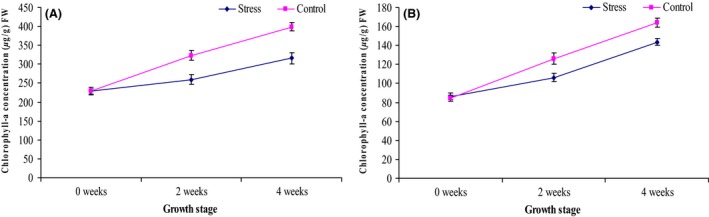
Average concentration of chlorophylls (A) chlorophyll a and (B) chlorophyll b of the stressed and control treatments of African eggplant accessions during different growth and development stages. Three independent leaf tissues were measured and expressed as mean ± SD (*μ*g/g) fresh weight. The leaves were sampled before stress (0 weeks), 2 and 4 weeks after stress.

**Figure 5:**
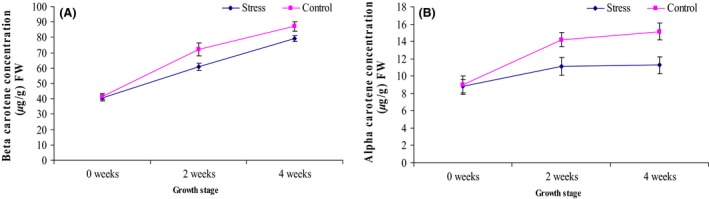
Average concentration of carotenes (A) *β*‐carotene and (B) *α*‐carotene of the stressed and control treatments of African eggplant accessions during different growth and development stages. Three independent leaf tissues were measured and expressed as mean ± SD (*μ*g/g) fresh weight. The leaves were sampled before stress (0 weeks), 2 weeks and 4 weeks after stress.

**Figure 6:**
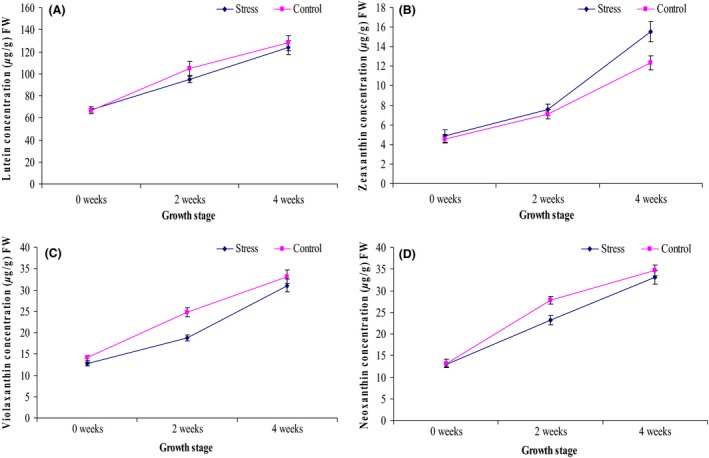
Average concentration of xanthophylls (A) lutein (B) zeaxanthin (C) violaxanthin and (D) neoxanthin of the stressed and control treatments of African eggplant accessions during different growth and development stages. Three independent leaf tissues were measured and expressed as mean ± SD (*μ*g/g) fresh weight. The leaves were sampled before stress (0 weeks), 2 and 4 weeks after stress.

The identified compounds were grouped into chlorophylls (chlorophyll a and chlorophyll b) (Fig. 4), carotenes (alpha carotene and beta carotene) (Fig. 5) and xanthophylls (lutein, zeaxanthin, violaxanthin and neoxanthin) (Fig. 6).

### Chlorophylls

The chlorophyll contents decreased with stress (Fig. 4) since a significantly higher (*P* < 0.05) concentration of chlorophylls was reported in the controls as compared to the drought stressed crops. Chlorophylls also increased with plant growth and development with mature leaves (4 weeks) reporting higher concentration.

### Carotenes

The concentration of carotenes; *β*‐carotene and *α*‐carotene are reported in Figure 5. Similar to the chlorophylls, a significant increase in the concentration was also seen as the crop grows. Likewise the trend was also the same as stressed crops had significantly lower concentration of carotene as compared to the controls. In addition, the *β*‐carotene was significantly higher (*P* < 0.05) as compared to *α*‐carotene.

### Xanthophylls

The concentration of xanthophylls lutein, zeaxanthin, violaxanthin and neoxanthin is reported in Figure 6. Contrary to the other xanthophylls, zeaxanthin reported increased concentration with the stress as compared to the control. Lutein had no significant difference between the stress and the control whereas violaxanthin and neoxanthin had significantly higher content in the control as compared to the stress.

## Discussion

In this study, a comparative analysis of the carotenoid and chlorophyll composition of leaves of 19 African eggplant accessions at different stages of maturity under adequate water availability or water stress conditions was done. Maturity in the leaves reflected enhanced carotenoid metabolic activities occurring during plant growth and development. This is reported by results showing changing concentrations with progressive plant growth and development as well as differences between in the stressed and control crops. There were significant differences in carotenoid concentration among the accessions used in the study. The concentration of all the carotenoids increased with growth and development of the plant. The most significant increase in the pigment concentration was recorded with 4 weeks after transplanting. This clearly indicates that the plant pigments accumulate as the plant grows. Some carotenoids were markedly characteristic of some accessions such as RV100343 and RV100342 which reported significantly higher contents (*P* < 0.05) of carotenoids as compared to the other accessions. These two accessions had a characteristic morphology of small leaf size as compared to the others.

Water stress, among other changes, has been reported to have the ability to reduce the tissue concentrations of chlorophylls and carotenoids (Kiani et al. 2008), primarily with the production of reactive oxygen species in the thylakoids (Reddy et al. 2004). The data from the study showed that the photosynthetic pigments such as the chlorophylls significantly reduced with stress. In general, the results obtained from this study agree with those reported in the literature and the values fall within the wide ranges of data found in the literature.

The variations in the levels of the major carotenoids lutein, *β*‐carotene and neoxanthin, were similar to those observed for the chlorophylls, with decreases observed under water stress. The major carotenoids, violaxanthin and *β*‐carotene increased progressively in control, whereas the proportion of lutein did not significantly change during the stress treatment. This is an indication that lutein is not significantly affected by water stress and this is an important avenue in the study of plan tolerance to stress. Variations similar to those of chlorophyll have also been observed for lutein, b‐carotene and neoxanthin in rosemary plants (Munné‐Bosch and Alegre 2000).

The proportions of zeaxanthin, neoxanthin, and violaxanthin were largely altered by the water stress treatment. During the stress treatment zeaxanthin increased whereas violaxanthin and neoxanthin decreased. On the contrary, RV100201, RV100432 and RV100342 accessions reported significantly higher carotenoids at 2 weeks and the contents reduced as the plant grows at 4 weeks. The fact that mature leaves suffered more stress than young leaves in these accessions suggests that developmental stages of these leaves might contribute to the differential prevention of oxidative damage in mature plants.

The decrease in chlorophyll content under drought is a commonly observed phenomenon (Heba and Samia 2014). This confirms the statement by Farooq et al. (2009) that both chlorophyll a and b are prone to soil drying. A reduction in chlorophyll content has also been reported in drought stressed cotton (Massacci et al. 2008) and Catharanthus roseus (Jaleel et al. 2009). In addition, the findings concur with that of the study by Kumar et al. (2011) who reported that chlorophyll content decreases under drought stress. This is because drought stress has been reported to cause closing of stoma, limitation of gas exchange and reduction of leaf area (Jaleel et al. 2009; Kumudini 2010); consequently decreasing photosynthetic pigments and activity. Similarly, this might be attributed to reduced synthesis of the main chlorophyll pigment complexes encoded by the cab gene family (Nikolaeva et al. 2010), or to destruction of the pigment protein complexes which protect the photosynthetic apparatus, or to oxidative damage of chloroplast lipids and proteins, therefore formation of chlorophyll a, b and other carotenoids decreases. Therefore, both stomatal and non‐stomatal limitations (metabolic impairment) are generally accepted to be the main determinant of reduced photosynthetic pigments under drought stress (Farooq et al. 2009).

A concerted action of both enzymatic (ascorbate peroxidase, superoxide dismutase, peroxidase, ascorbate peroxidase, catalase, polyphenol oxidase and glutathione reductase) and non‐enzymatic antioxidant (ascorbate, reduced glutathione, *α*‐tocopherol and carotenoids) systems alleviates oxidative damage generated by drought stress in the plant tissue (Prochazkova et al. 2001). Nevertheless, carotenes form a key part of the plant antioxidant defense system, but they are very susceptible to oxidative destruction. In severe stress, *β*‐carotene may be rapidly destroyed and therefore are no longer available to protect against oxidative damage (Young and Britton 1990). This explains the significant reduction in the concentrations of the stressed crops from the study.

The concentration of carotenoids and chlorophylls provide information about the level of stress experienced by the plant as well as its ability to endure these stresses (Strzalka et al. 2003). Therefore, due to the significant decrease in carotenoids during drought stress, it is evident that drought may lead to reduction in plant productivity. This is mainly by inhibiting growth and photosynthesis, and is one major limiting factor in agriculture worldwide leading to huge reductions in crop yield (Chaves and Oliveira 2004).

## Conclusion

This study focused on the quantification of carotenoids of the leaves of African eggplants commonly consumed as leafy and fruit vegetables. The results gave comparative profiles of carotenoids at different growth and developmental stages and under drought stress. Perhaps the carotenoid levels increase with plant maturity, therefore in any case, mature leaves can provide high amounts of carotenoids that are important to human health. As well water stress affects the levels of carotenoids especially the chlorophylls which are the primary photosynthetic pigments. With this it is evident that plants subjected to drought stress have reduced levels of plant pigments such as chlorophylls and carotenes and increased xanthophylls. This adversely affects the functioning of the plant as it hinders the process of photosynthesis. Some of the observed carotenoid compositional changes could directly be related to known phenomenon associated with plant development, stress and photosynthetic activity. These changes may not only affect the nutritional value of the leaves abut also the health and medicinal value of the crops. The study therefore, presents data that attests to the importance of African eggplants in providing the much‐needed dietary neutraceutical potential since they have substantial amounts of the important carotenoids. There is need to characterize other secondary metabolites of medicinal value such as the flavonoids, anthocyanins so as to state the effects of stress on these. This will help define the effect of stress on different plant metabolites and might also help in study of stress tolerance in plants.

## Conflict of Interest

None declared.
